# Dynamic Interferometry for Freeform Surface Measurement Based on Machine Learning-Configured Deformable Mirror

**DOI:** 10.3390/s25020490

**Published:** 2025-01-16

**Authors:** Xu Chang, Yao Hu, Jintao Wang, Xiang Liu, Qun Hao

**Affiliations:** 1 Institute of Mechanics and Acoustics Metrology, National Institute of Metrology, Beijing 100029, China; liuxiang@nim.ac.cn; 2 Beijing Key Laboratory for Precision Optoelectronic Measurement Instrument and Technology, School of Optics and Photonics, Beijing Institute of Technology, Beijing 100081, China; huy08@bit.edu.cn (Y.H.); qhao@bit.edu.cn (Q.H.); 3 School of Opto-Electronic Engineering, Changchun University of Science and Technology, Changchun 130022, China

**Keywords:** dynamic interferometry, freeform surface, machine learning

## Abstract

Optical freeform surfaces are widely used in imaging and non-imaging systems due to their high design freedom. In freeform surface manufacturing and assembly, dynamic freeform surface measurement that can guide the next operation remains a challenge. To meet this urgent need, we propose a dynamic interferometric method based on a machine learning-configured deformable mirror (DM). In this method, a dynamic interferometric system is developed. By using coaxial structure and polarization interference, transient measurement of the measured surface can be realized to meet dynamic requirements, and at the same time, DM transient monitoring can be realized to reduce the accuracy loss caused by DM surface changes and meet dynamic requirements. A transient phase modulation scheme using machine learning to configure the DM surface is proposed, which keeps the system in a measurable state. Compared with the traditional phase modulation scheme that relies on iteration, the scheme proposed in this paper is more efficient and is conducive to meeting dynamic requirements. The feasibility is verified by practical experiments. The research in this paper has significance for guiding the application of dynamic interferometry in the measurement of dynamic surfaces.

## 1. Introduction

The optical freeform surface can significantly improve system performance and promote lightweight equipment due to its non-rotationally symmetric structure with high design freedom [1,2]. Benefiting from its advantages, freeform surfaces are widely used in imaging optical systems, such as virtual reality and augmented reality head-mounted displays [3,4], off-axis reflection systems [5,6], and ultraviolet lithography systems [7,8], as well as non-imaging optical systems, such as lighting [9], laser beam shaping [10], etc.

The application of freeform surfaces in the above-mentioned optical systems is attributed to the development of optical manufacturing, coating, and assembly technology. Among these technologies, there is a demand for dynamic freeform surface measurement. Firstly, the manufacturing process of optical freeform surfaces includes rough machining stages such as grinding and milling, as well as precision machining stages such as polishing. In the rough machining stage, there is a significant difference between the machined surface and the theoretical surface, and dynamic measurement is not necessary to provide timely guidance. Static measurement can meet the measurement requirements of this stage. In the critical precision machining stage, the machined surface approaches the theoretical surface, and its surface roughness reaches the nanometer level. Manufacturing errors caused by external disturbances can lead to deviations between the actual surface and the theoretical surface [11,12,13], which need to be dynamically measured to guide the next process. However, most of the measurement during precision machining is aimed at the static surfaces that leave the production line, resulting in low feedback efficiency. A method is needed to measure the dynamic surface that changes during manufacturing to guide the next step. Secondly, freeform surfaces require coating due to application scenarios. The difference in thermal expansion between the film material and the freeform surface substrate can cause film stress, which can lead to surface figure error and affect surface accuracy. At present, the research on the influence of film stress on freeform surface figure error is mostly focused on the stages after film growth and annealing [14,15]. A method is needed to measure the changing dynamic surface during film growth, which can be used to improve the physical models of film stress and strain to predict surface shape and improve yield rate. Finally, in the precise assembly of freeform surfaces, since the freeform surface achieves lightweight structures through the diversity of surface shapes, the stress generated by the clamping tool can easily lead to dynamic changes in the surface shape as the clamping tool is adjusted. This phenomenon is not conducive to the performance of optical systems that require high surface accuracy. A method is needed to measure the dynamic freeform surfaces to guide assembly.

The dynamic surface measurement needs to meet the requirements of dynamic and accuracy. At present, the measurement methods of freeform surfaces mainly include non-interferometry [16,17,18] and interferometry [19,20,21]. Interferometry has the potential to meet these requirements due to its advantages of non-contact, fast measurement speed, and high precision. The compensation interferometry uses a compensator to obtain a resolvable full-aperture interferogram at one time, putting the system in a measurable state, and the measurement can be completed quickly. However, the compensation phase provided by conventional compensators such as lenses, lens groups, mirrors, and computer-generated holograms [22,23,24] is fixed rather than dynamic as required for dynamic measurements. A deformable mirror (DM) that can generate variable surfaces can be adopted by interferometric systems to provide dynamic phases [25].

The interferometry using a DM for dynamic measurement needs to meet the following requirements: (1) To meet dynamic requirements, the surface under test (SUT) needs to be measured transiently. At the same time, in order to meet the accuracy requirements, the DM’s surface needs to be transiently monitored while measuring the SUT. This is because the DM’s surface, which is used to calculate the measurement results, changes during the measurement of the SUT due to factors such as hysteresis and creep, which is not conducive to accuracy. The reason why monitoring requires transient implementation is to meet dynamic requirements. (2) Phase modulation can be realized transiently. In dynamic measurement, the required phase of the system should change with SUT to keep the system in a measurable state. Thus, the phase modulation needs to be realized transiently by controlling the DM’s surface.

In the earlier literature, the interferometry using the DM makes it difficult to meet dynamic requirements and maintain satisfactory accuracy at the same time. Fuerschbach et al. [26] proposed an interferometric method for measuring a known φ polynomial mirror surface. A DM was used, and its surface was generated and measured by a configuration independent of the measurement system before SUT measurement. Therefore, the measurement of SUT and DM was not simultaneous, making it difficult to meet dynamic requirements.

Huang et al. [27] proposed an adaptive interferometric null testing method for unknown freeform surface measurement. This method adopted DM to achieve phase modulation. The DM surface was optimized by an optimization algorithm, and a DS system [28] was built to monitor the DM. This method can realize unknown freeform surface measurements without generating the DM’s surface in advance and improves the accuracy through DM monitoring. However, multiple patterns typically need to be projected in the monitoring. Therefore, it is difficult to realize transient monitoring and meet dynamic requirements.

Zhang et al. carried out a series of studies on freeform surface measurement methods using a DM. Firstly, an adaptive interferometer for freeform surfaces was proposed [29]. The DM’s surface was obtained through iterative optimization of commercial adaptive software. The measurement system contained two charge-coupled devices (CCD), one for SUT measurement and the other for DM monitoring. The coaxial optical path can realize measurement and monitoring simultaneously, which is conducive to accuracy. However, the measurement and monitoring of this method were realized by time-consuming mechanical phase shifting, which is not conducive to dynamic requirements. Subsequently, Zhang et al. proposed a model-based adaptive non-null interferometry for freeform surface measurement [30]. For a static SUT, multiple DM surfaces that can achieve non-null compensation were generated and monitored by wavefront sensors, and multiple resolvable system interferograms corresponding to each DM surface were obtained by a CCD. A multi-configuration ray-tracing algorithm was established to obtain figure errors. In this method, DM monitoring is conducive to accuracy. However, this method still relies on mechanical phase shifting, and the process of the DM generating multiple surfaces is time consuming, which means that this method finds it difficult to meet dynamic requirements. Based on the previous study, Zhang et al. proposed a compact interferometer with two DMs [31] and an interferometer with an adaptive ring-cavity compensator [32]. The measurement range of these interferometers was expanded, and the freeform surface with large departure can be measured. However, measurement and monitoring were realized sequentially through one CCD, and so they were not simultaneous. Thus, the dynamic performance of these two methods is limited.

Transient phase modulation plays an important role in dynamic measurement. The phase modulation in the methods proposed in Refs. [27,29,31] was realized by optimizing the DM’s surface through iterative optimization algorithms such as the stochastic parallel gradient descent (SPGD) algorithm [33], iterative adaptive algorithm, and the algorithm combining SPGD and influence function matrix. However, iterative optimization algorithms find it difficult to realize transient phase modulation due to the following reasons: (1) Iterative optimization is time consuming, so the efficiency is limited. (2) The feedback of the iteration is usually the interferogram corresponding to the SUT. Dynamic changes in the SUT can lead to non-convergence, so the phase modulation may not be realized.

The above existing methods using DM can be divided into the following two categories: One category is that SUT measurement and DM monitoring are not simultaneous. This category mainly includes the method proposed in Refs. [26,31,32]. The other category is that SUT measurement and DM monitoring are simultaneous but time consuming. This category mainly includes the method proposed in Refs. [27,29,30]. These two categories of methods are time consuming, so it is difficult to meet the dynamic requirements. In addition, most of these methods used time-consuming iterative optimization algorithms to optimize the DM’s surface to achieve phase modulation, therefore making it difficult to meet the dynamic requirements.

According to the above, in order to meet the dynamic requirements and maintain satisfactory accuracy, firstly, SUT measurement and DM monitoring need to be simultaneously and transiently realized. Secondly, an efficient phase modulation method that can be realized transiently is needed. In this paper, the concepts of transient and dynamic are not equal. Transient focuses on the measurement of a certain surface generated during the dynamic SUT change process, completing phase modulation, measurement, and monitoring operations at certain moments. Dynamic focuses on the entire process of dynamic SUT measurement, completing a series of surface measurements. Dynamic is based on transient.

To achieve these goals, we proposed a dynamic interferometry for freeform surface measurement based on a machine learning-configured deformable mirror and we named it DFMD. D stands for dynamic, F stands for freeform surface, M stands for machine learning, D stands for deformable mirror. In DFMD, a dynamic interferometric system with DM monitoring is designed, which uses coaxial structure and polarization optics to simultaneously realize SUT transient measurement and DM transient monitoring to meet dynamic and accuracy requirements. A transient phase modulation scheme based on a machine learning-controlled DM is proposed. In this scheme, the target phase of modulation is transiently realized by a DM surface control neural network model. DFMD implements measurement based on a dynamic measurement strategy that combines the dynamic interferometric system and the transient phase modulation scheme. To verify the feasibility of DFMD, practical experiments are carried out.

This paper is organized as follows: Section 2 introduces the principle of DFMD, including the dynamic measurement strategy, the dynamic interferometric system with DM monitoring, and the transient phase modulation scheme based on a machine learning-controlled DM. Section 3 shows the practical experiments and discussions used to verify the feasibility of DFMD. Section 4 shows the conclusion.

## 2. Methods and Theory

In DFMD, measurements are carried out according to the dynamic measurement strategy that combines the dynamic interferometric system with DM monitoring and the transient phase modulation scheme based on machine learning-controlled DM. In this interferometric system, a DM is adopted to realize dynamic phase modulation, and a coaxial structure and polarization optics are used to design the system structure. SUT transient measurement and DM transient monitoring can be realized at the same time through polarization phase shifting. In this phase modulation scheme, the target phase of modulation is obtained transiently and directly through the dynamic interferometric system and is realized transiently by generating the corresponding DM surface through the DM surface control neural network model.

### 2.1. Dynamic Measurement Strategy

**Figure 1:**
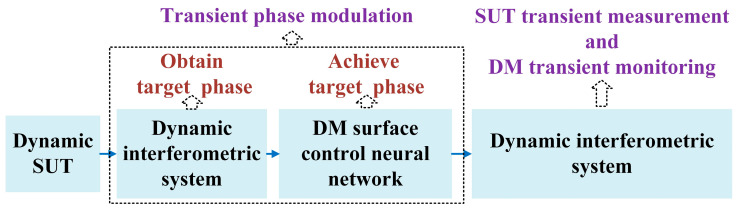
Flowchart of a single measurement.

The SUTs of dynamic measurement are series of gradually changing surfaces corresponding to certain moments, which is consistent with the changing trend of measured surfaces in practical applications. Each SUT can be measured according to the single measurement shown in Figure 1, which combines the dynamic interferometric system described in Section 2.2 and the transient phase modulation scheme described in Section 2.3. DFMD implements dynamic measurement by repeating a single measurement.

In a single measurement, firstly, the dynamic interferometric system is used to obtain the target phase. Secondly, the DM surface control neural network model is used to control the DM surface to achieve target phase modulation. Finally, the dynamic interferometric system is used to simultaneously realize SUT transient measurement and DM transient monitoring.

### 2.2. Dynamic Interferometric System with Deformable Mirror Monitoring

**Figure 2:**
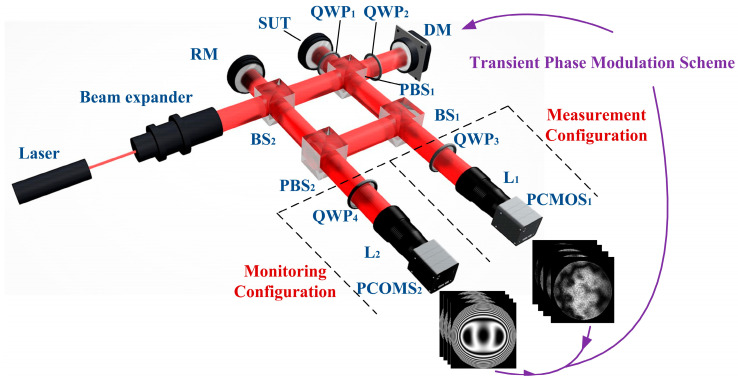
Layout of dynamic interferometric system with DM monitoring in DFMD.

Figure 2 shows the layout of the dynamic interferometric system with DM monitoring that we designed, inspired by the literature [29,30,31,32]. To express clearly, the devices in Figure 2 are abbreviated. The full spelling of the abbreviations is shown in the next paragraph. For the convenience of assembly, the system is based on the coaxial structure of the Twyman–Green interferometer, and the DM is adopted as the reference surface. The DM’s surface in the measurement is changed with the dynamic SUT, which is realized by the phase modulation scheme described in Section 2.3. To simultaneously realize SUT transient measurement and DM transient monitoring, the measurement configuration and the monitoring configuration are constructed by using polarizing optical elements, respectively. The polarization property of the beam is changed to realize polarization phase-shifting interference. Each of these two configurations uses a polarization complementary metal oxide semiconductor camera (PCMOS). Two PCMOSs are controlled simultaneously, and they can all obtain an original image transiently. The original image can be decomposed into four polarization phase-shifting interferograms to calculate the measurement results.

**Figure 3:**
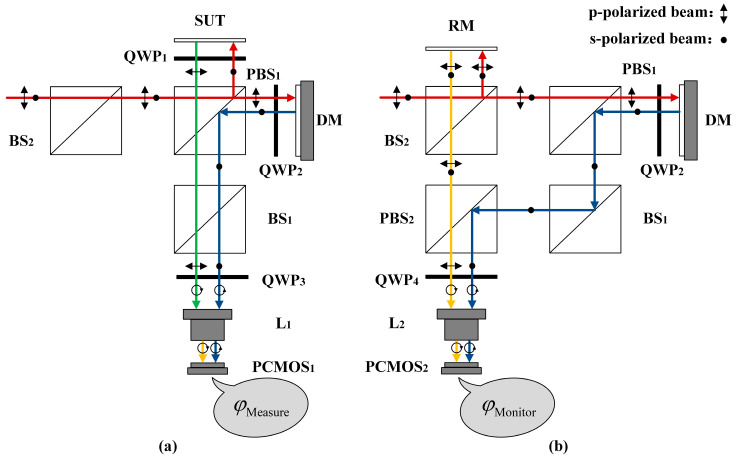
Layout of (**a**) measurement configuration and (**b**) monitoring configuration.

Figure 3a,b show the layout of the measurement configuration and monitoring configuration, respectively. In Figure 3a, the red solid lines represent the incident beams incident on SUT and DM. Specifically, the circularly polarized beam collimated by the beam expander is incident on a beam splitter (BS_2_) and then travels through a polarized beam splitter (PBS_1_). The p-polarized component and s-polarized component of this beam are transmitted and reflected, respectively, and become a p-polarized beam and an s-polarized beam. The p-polarized beam travels through a quarter-wave plate (QWP_2_) and is incident on the DM. The s-polarized beam travels through QWP_1_ and incident on SUT. The blue solid lines and green solid lines represent the beams reflected by the DM and SUT, respectively. The beam reflected by the DM travels through QWP_2_, PBS_1_, and BS_1_, and is transformed into an s-polarized beam. The beam reflected by the SUT travels through QWP_1_, PBS_1_, and BS_1_, and is transformed into a p-polarized beam. The s-polarized beam and p-polarized beam travel through QWP_3_ and L_1_, and are transformed into circularly polarized beams with opposite rotation. The circularly polarized beams travel through the imaging lens (L_1_) and incident on PCMOS_1_. An original image is transiently obtained by PCMOS_1_, and four polarization phase-shifting interferograms corresponding to the polarization directions of 0°, 45°, 90°, and 135° can be extracted from the original image to calculate the phase of measurement configuration $\varphi_{\mathrm{Measure}}$, which is generated owing to the surface shape difference between SUT and DM. If the retrace error can be ignored, the surface figure error of SUT relative to the DM can be expressed as follows:

(1) $$e_{\mathrm{SFE}\_\mathrm{SUT}}=\frac{1}{2}\cdot \frac{1}{n_{0}}\cdot \frac{\lambda }{2\pi }\cdot \varphi_{\mathrm{Measure}},$$

where $n_{0}=1$ is the refractive index of air and λ = 632.8 nm is the wavelength of the laser light source in the interferometric system.

In Figure 3b, the red solid lines represent the incident beams incident on the reference mirror (RM) and DM. Specifically, the circularly polarized beam collimated by the beam expander is split into two parts by BS_2_. The transmitted beam is transformed into a p-polarized beam after traveling through PBS_1_, and it travels through QWP_2_ and is incident on DM. The reflected beam is incident on RM. The blue solid lines and yellow solid lines represent the beams reflected by DM and RM, respectively. The beam reflected by the DM travels through QWP_2_, PBS_1_, BS_1_, and PBS_2_, and is transformed into an s-polarized beam. The beam reflected by the RM travels through BS_2_ and PBS_2_ and is transformed into a p-polarized beam. The s-polarized beam and p-polarized beam travel through QWP_4_ and L_2_ and are incident on PCMOS_2_. Similar to PCMOS_1_, the phase of monitoring configuration $\varphi_{\mathrm{Monitor}}$ can be calculated, which is generated owing to the surface shape difference between RM and DM. In the case of ignoring system errors and RM being an ideal plane, $\varphi_{\mathrm{DM}}=\varphi_{\mathrm{Monitor}}$, where $\varphi_{\mathrm{DM}}$ is the phase generated by DM. The surface figure error of DM relative to RM $e_{\mathrm{SFE}\_\mathrm{DM}}$ is equal to the DM’s surface $e_{\mathrm{DM}}$:

(2) $$e_{\mathrm{DM}}=e_{\mathrm{SFE}\_\mathrm{DM}}\approx \frac{1}{2}\cdot \frac{1}{n_{0}}\cdot \frac{\lambda }{2\pi }\cdot \varphi_{\mathrm{Monitor}}.$$

The measurement results of SUT can be calculated by combining Equations (1) and (2). This method focuses on the relative difference of continuous surfaces. Superimposing or subtracting integer multiples of 2π on the calculated phase can be considered as an overall translation. Translation does not affect the relative difference. Therefore, 2π ambiguity is not considered in this method.

### 2.3. Transient Phase Modulation Scheme Based on Machine Learning-Controlled DM

In the measurement configuration shown in Figure 3a, the phases of the beams from the SUT and DM incident on PCMOS_1_ are the measurement phase and the reference phase, respectively. These two phases should be consistent so that the measurement configuration (PCMOS_1_) can obtain a resolvable sparse interferogram. The measurement phase changes with SUT, so the reference phase needs to be modulated. In DFMD, the phase modulation needs to be realized transiently to meet dynamic requirements. To achieve this goal, a transient phase modulation scheme based on a machine learning-controlled DM is proposed, which includes two parts.

First, the target reference phase $\varphi_{\mathrm{Target}}$ is obtained transiently and directly through the dynamic interferometric system shown in Figure 2, instead of clarifying the optimization direction based on the sparsity of interference fringes in the iterative optimization method. $\varphi_{\mathrm{Target}}$ consists of two parts: one part is the phase generated by DM $\varphi_{\mathrm{DM}}$, which is considered equal to $\varphi_{\mathrm{Monitor}}$; and the other part is the difference between the phases generated by SUT and DM, which is the phase of the measurement configuration $\varphi_{\mathrm{Measure}}$. PCMOS_1_ and PCMOS_2_ are simultaneously controlled to obtain the original image for calculating $\varphi_{\mathrm{Measure}}$ and $\varphi_{\mathrm{Monitor}}$, and $\varphi_{\mathrm{Target}}=\varphi_{\mathrm{Measure}}+\varphi_{\mathrm{Monitor}}$ is obtained. $\varphi_{\mathrm{Target}}$ can be transiently obtained because PCMOS takes milliseconds to obtain an image and complete the calculation.

Second, $\varphi_{\mathrm{Target}}$ is transiently realized through the DM surface control neural network model, rather than through a time-consuming iterative optimization algorithm. $\varphi_{\mathrm{Target}}$ can be realized by controlling DM to generate a target surface $e_{\mathrm{DM}\_T}$. Without considering the retrace error, the relationship between $\varphi_{\mathrm{Target}}$ and the DM’s target surface $e_{\mathrm{DM}\_T}$ can be expressed as follows:

(3) $$\varphi_{\mathrm{Target}}\approx 2\cdot n_{0}\cdot \frac{2\pi }{\lambda }e_{\mathrm{DM}\_T}.$$

**Figure 4:**
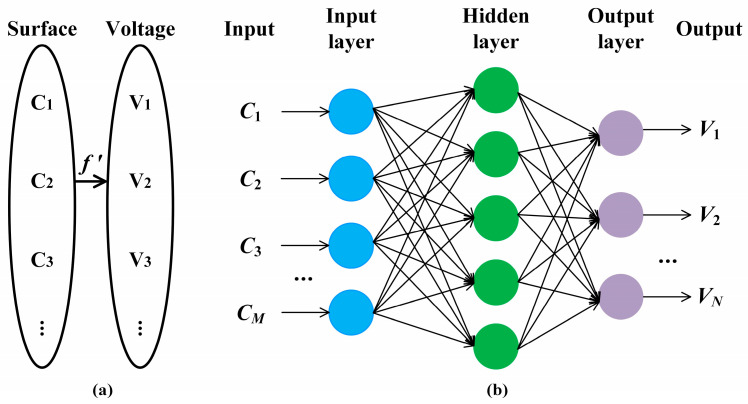
(**a**) Non-linear one-to-one mapping relationship $f^{\prime }$ between **C** and **V**, and (**b**) the structure layout of the DM surface control neural network model.

The DM surface is controlled by the control voltage of each piezoelectric actuator (PZT), and the non-linear one-to-one mapping relationship $f^{\prime }$ between them is shown in Figure 4a. $C=\left[c_{1},c_{2},c_{3},\cdots ,c_{M}\right]$ is the DM surface vector formed by the coefficients obtained by fitting $e_{\mathrm{DM}\_T}$ using Zernike standard polynomials. *M* is the number of terms of Zernike standard polynomials. Since the DM surface is low-frequency information, using **C** to represent the DM surface is conducive to reducing the data dimension. $V=\left[v_{1},v_{2},v_{3},\cdots ,v_{N}\right]$ is the control voltage vector of PZTs corresponding to **C**, and *N* is the number of PZTs. $C_{i}$ is the *i*-th DM surface vector, and its corresponding control voltage vector is $V_{i}$, *i* = 1, 2, 3, …. If $f^{\prime }$ is constructed, **V** can be calculated according to **C**, and the DM target surface $e_{\mathrm{DM}\_T}$ can be generated by loading **V** to realize the target reference phase $\varphi_{\mathrm{Target}}$.

Based on the earlier literature, $f^{\prime }$ can be constructed based on the influence function matrix. **V** can be directly calculated according to the inverse matrix of the influence function [34]. This method is efficient, but there are linear approximations in the establishment of the influence function matrix, so the DM surface control accuracy is limited. Furthermore, **V** can be optimized by the influence function matrix [35,36]. However, this method relies on iterative optimization, resulting in unsatisfactory efficiency, and therefore it cannot generate DM target surface transiently.

In this paper, $f^{\prime }$ is constructed by building a DM surface control neural network model as shown in Figure 4b, which adopts a fully connected neural network. Fully connected neural networks and convolutional neural networks are two typical types of neural networks. Among them, convolutional neural networks are suitable for situations with high input dimensions and are good at solving image-related problems. Compared with convolutional neural networks, fully connected neural networks have a simpler structure, faster learning speed, and are more suitable for processing low-dimensional data types. Since the surface shape of DM is low-frequency information, the DM surface vector **C** formed by the characterization parameters obtained by Zernike fitting is a one-dimensional data point, and the control voltage vector **V** is a one-dimensional data point. Therefore, this paper uses a fully connected neural network to establish the mapping relationship $f^{\prime }$. The feasibility of fitting nonlinear mapping relationships by the neural network is verified in references [25,37,38].

In Figure 4b, the input is the elements of surface vector **C**, $c_{1}$ to $c_{M}$. The number of neurons in the input layer is equal to the input dimension *M*. The number of hidden layers and neurons in each layer is determined by network training, and this figure is for illustration only. The number of neurons in the output layer is *N*, which is equal to the dimension of the output control voltage vector **V**. The output is the elements of control voltage vector **V**, $v_{1}$ to $v_{N}$. The following process takes about a few milliseconds: 

(1) $e_{\mathrm{DM}\_T}$ is fitted to obtain **C**. 

(2) **C** is input into the neural network model to calculate the predicted control voltage vector. 

(3) The predicted control voltage vector is loaded onto the DM to generate the corresponding surface. Therefore, the phase modulation can be completed transiently.

## 3. Practical Experiments and Discussions

To verify the feasibility of DFMD, practical experiments were carried out. Firstly, a DM surface control neural network model was constructed and its control accuracy was tested. Secondly, a dynamic interferometric experimental system was built and the system errors were analyzed and calibrated. Subsequently, accuracy and dynamic experiments were carried out to verify the feasibility of the accuracy and dynamics, respectively. Finally, the factors affecting the dynamic response were analyzed. We did not take special temperature and humidity control measures. The ambient temperature and relative humidity during the experiment were in the range of 20 ± 2 °C and 45 ± 5%, respectively.

### 3.1. Deformable Mirror Surface Control Neural Network Model

The DM adopted in this paper is PAD-2-300-64 piezoelectric deformable mirror produced by Visionica company, its aperture diameter is 50 mm, and the number of PZTs is *N* = 52. The DM surface can be controlled according to the “Group mode” provided by the manufacturer. “Group mode” includes 26 Groups, such as Group_1, Group_2, etc., and each Group corresponds to an aberration surface. In “Group mode”, the control voltage of each PZT is the product of the total control voltage and its corresponding weight parameter.

The DM surface control neural network model was constructed through network training. To obtain diverse DM surface samples for training, Group_1 to Group_26 were selected sequentially, and the value of the total control voltage was set in steps of 2V within the effective range. The control voltage vectors **V** were recorded, and the corresponding DM surfaces were measured by Zygo interferometer produced by ZYGO company in Middlefield, United States. The sampling points of DM surface within the 20 mm aperture obtained by the Zygo interferometer were fitted by the first 153 terms of the Zernike standard polynomial. Only the coefficients of the 4th to 153rd polynomials were retained to form the DM surface shape vector **C**, therefore *M* = 150. The use of 153 terms instead of the more widely used 37 terms or higher was to improve the fitting error while reducing the computational complexity. The total number of DM surface samples was 6787. According to the equal interval strategy, the samples were divided into training set, verification set, and test set, and the number of samples in each set was 5897, 170, and 670, respectively.

In the neural network model training, the input and output dimensions were 150 and 52, respectively, the number of hidden layers was 5, and the number of neurons in each hidden layer obtained by genetic algorithm optimization was 148, 117, 115, 85, and 80, respectively. The normalization function was BatchNorm, the loss function regularization method is L2 regularization, and the regularization parameter was 0.007. The weight parameter initialization method was normal distribution, and its standard deviation was 0.722. The initial value of the learning rate was 0.005, the learning rate optimization criterion was RMSprop, the batch training size was 128, and the number of training times was 200.

**Figure 5:**
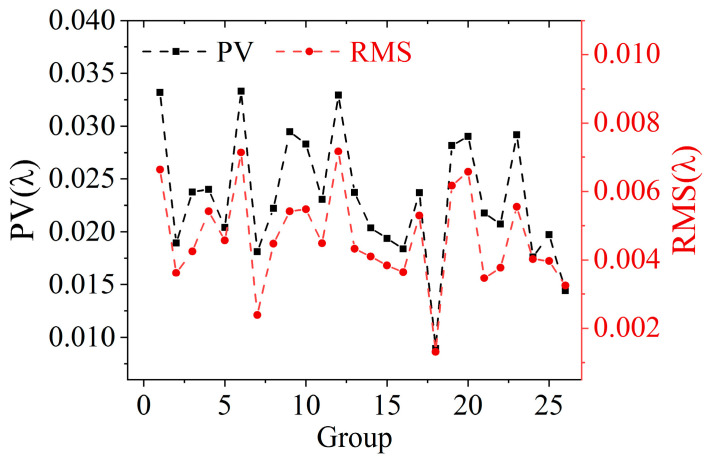
PV and RMS of the deviation between the DM predicted surface and the DM sample surface for 26 samples belonging to Group_1 to Group_26, respectively.

The control accuracy of this model was tested by 26 samples, which were selected from the test set and belonged to Group_1 to Group_26, respectively. The surface vector **C** of each sample was input into the model to obtain the predicted control voltage vector **V_pred_**, which was loaded and the corresponding DM predicted surface was measured by Zygo interferometer. The control accuracy can be illustrated by the deviation between the DM predicted surface and the DM sample surface. Considering that the reproducibility of the measurement environment affects control accuracy, the DM sample surface was obtained by loading the control voltage vector **V** of the sample. Figure 5 shows the peak to valley (PV) and root mean square (RMS) of the deviation between the DM predicted surface and the DM sample surface for 26 samples. The black dashed line is the PV curve, and the red dashed line is the RMS curve. The maximum and minimum values of PV are 0.0333 λ and 0.0090 λ, respectively, and the maximum and minimum values of RMS are 0.0072 λ and 0.0013 λ, respectively, and λ = 632.8 nm. There is no obvious trend in the fluctuations of PV and RMS of samples corresponding to different groups. According to the above, the control accuracy of the DM surface control neural network model reaches nanometers in both PV and RMS.

In addition to comparing PV and RMS, the control effect of this model can also be evaluated by mean absolute error (MAE) value. The MAE value is calculated according to the control voltage vector **V** of the sample and the predicted control voltage vector **V**_pred_, and its expression is as follows:

(4) $$\mathrm{MAE}=\frac{\sum_{n=1}^{N}\left|V_{\mathrm{pred},n}-V_{n}\right|}{N}$$

**Figure 6:**
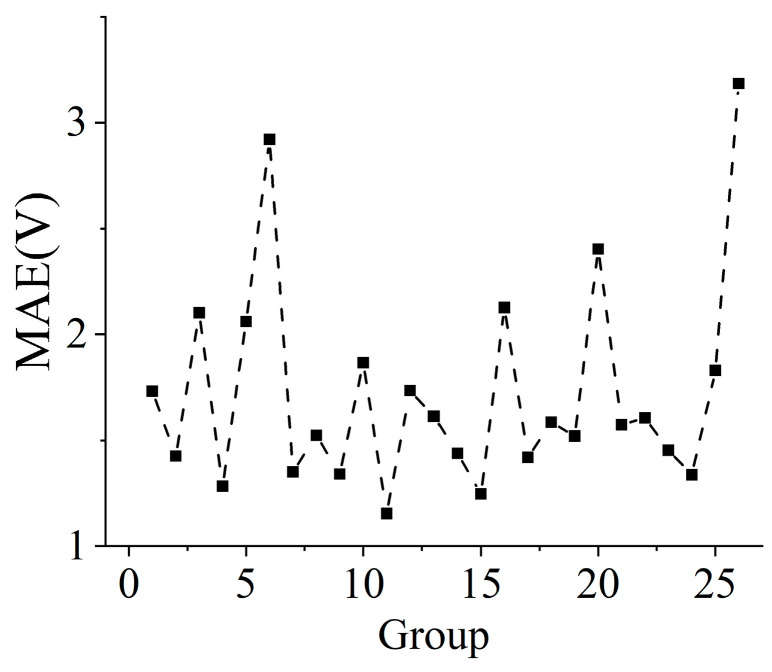
MAE of 26 samples belonging to Group_1 to Group_26, respectively.

The smaller the MAE, the closer **V**_pred_ is to **V**, and the better the control effect. Figure 6 shows the MAE value of 26 samples belonging to Group_1 to Group_26. In Figure 6, the maximum MAE value is 3.18 V corresponding to Group_26, and the minimum value is 1.16 V corresponding to Group_11. The MAE of different samples shows obvious fluctuations, and there is no obvious change trend between them.

### 3.2. Dynamic Interferometric Experimental System and System Error Calibration

**Figure 7:**
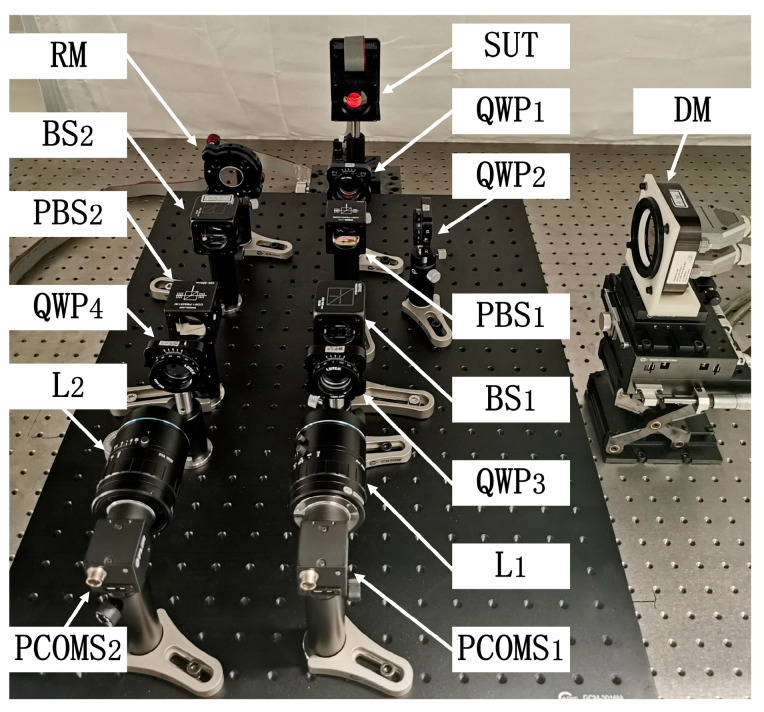
Layout of the dynamic interferometric experimental system.

A dynamic interferometric experimental system was built, as shown in Figure 7. The collimated circularly polarized beam with a wavelength of λ = 632.8 nm provided by the Zygo interferometer was used as the incident light. The splitting ratio of BS_1_ and BS_2_ was 5:5, RM, QWP_1_, QWP_2_, QWP_3_, and QWP_4_ were provided by LBTEX company in Changsha, China, and PBS_1_ and PBS_2_ were provided by Thorlabs. The HF3514V imaging lenses provide by u-Tron company in Tokyo, Japan were adopted as L_1_ and L_2_. FLIR BFS-U3-51S5P-C with a resolution of 2048 pixels × 2448 pixels was used as PCMOS_1_ and PCMOS_2_. The DM used in this system was the same one described in Section 3.1. The SUTs were generated by a piezoelectric deformable mirror (PDM), which is produced by OKO company in Rijswijk, Netherlands with an aperture of 30 mm. The measured aperture of PDM was 20 mm.

In phase calculation, the pixels of the four polarization phase-shifting interferograms extracted from the original image of PCMOS_1_ and PCMOS_2_ were 1024 × 1224 pixels. Therefore, the resolution of the current experimental system is 1024 pixels × 1224 pixels in principle. The resolution of the measured surface is about 0.016 mm. Without changing the PCMOS used in the system, the change in aperture does not affect the resolution of the experimental system, and the change in aperture is proportional to the resolution value of the measured surface. For the measurement requirements of large apertures with high resolution, it is necessary to replace the PCMOS with a higher resolution than the one used in this paper.

In the process of building and assembling the experimental system, to reduce the influence of optical mixing, we selected high-performance polarization optical elements and used PAX1000VIS produced by Thorlabs in Newton, United States to strictly control the polarization state of the beam. The interferograms shown in Section 3.2, Section 3.3 and Section 3.4 did not find obvious overlapping interference fringes, and the comparison of the measurement results of this experimental system and Zygo in Section 3.2 shows the acceptable accuracy of this method.

To obtain reliable measurement results, the system error of the experimental system was analyzed and calibrated. The system error includes two parts: phase $\varphi_{\mathrm{SPE}}^{\mathrm{Monitor}}$ and $\varphi_{\mathrm{SPE}}^{\mathrm{Measure}}$ introduced by the manufacturing error of the optical elements in the monitoring configuration and the measurement configuration, respectively. In the experimental system, the phase $\varphi_{\mathrm{Monitor}}$ of the monitoring configuration and the phase $\varphi_{\mathrm{Measure}}$ of the measurement configuration are, respectively, expressed as follows:

(5) $$\varphi_{\mathrm{Monitor}}=\varphi_{\mathrm{DM}}+\varphi_{\mathrm{SPE}}^{\mathrm{Monitor}},$$

(6) $$\varphi_{\mathrm{Measure}}=\varphi_{\mathrm{SUT}}-\varphi_{\mathrm{DM}}+\varphi_{\mathrm{SPE}}^{\mathrm{Measure}}.$$

When $\varphi_{\mathrm{SPE}}^{\mathrm{Monitor}}$ and $\varphi_{\mathrm{SPE}}^{\mathrm{Measure}}$ are calibrated, the phase $\varphi_{\mathrm{DM}}$ generated by the DM and the phase $\varphi_{\mathrm{SUT}}$ generated by the SUT can be obtained according to Equations (5) and (6). When the theoretical surface $e_{\mathrm{SF}\_\mathrm{ideal}}$ of SUT is known, the surface figure error of the SUT can be expressed as follows:

(7) $$e_{\mathrm{SF}\_\mathrm{error}}=\frac{1}{2}\cdot \frac{1}{n_{0}}\cdot \frac{\lambda }{2\pi }\cdot \varphi_{\mathrm{SUT}}-e_{\mathrm{SF}\_\mathrm{ideal}}.$$

In order to analyze the accuracy of DFMD, the measurement results of DFMD and Zygo interferometer are compared in this paper. So, the surface figure error of SUT in this paper is relative to the plane, which is consistent with the Zygo interferometer.

**Figure 8:**
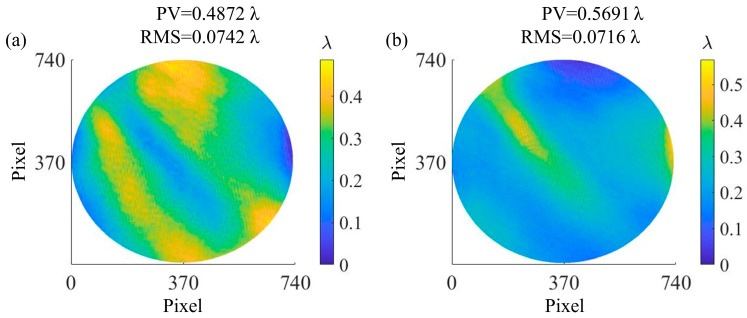
System error of (**a**) monitoring configuration $\varphi_{\mathrm{SPE}}^{\mathrm{Monitor}}$ and (**b**) measurement configuration $\varphi_{\mathrm{SPE}}^{\mathrm{Measure}}$.

In the calibration experiment of $\varphi_{\mathrm{SPE}}^{\mathrm{Monitor}}$, the DM in the experimental system was replaced by standard flat mirror 1 (SFM_1_), and PCMOS_2_ was used to obtain interferograms and calculate $\varphi_{\mathrm{SPE}}^{\mathrm{Monitor}}$ as shown in Figure 8a. The PV and RMS of $\varphi_{\mathrm{SPE}}^{\mathrm{Monitor}}$ are 0.4872 λ and 0.0742 λ, respectively. Similarly, in the calibration experiment of $\varphi_{\mathrm{SPE}}^{\mathrm{Measure}}$, the SUT was replaced by standard flat mirror 2 (SFM_2_), and PCMOS_1_ was used to obtain $\varphi_{\mathrm{SPE}}^{\mathrm{Measure}}$ as shown in Figure 8b, with a PV of 0.5691 λ and an RMS of 0.0716 λ. 

Without changing the optical elements used in the system, the system error does not change in theory. However, in order to cope with the possible changes in the optical elements during online measurement due to factors such as ambient temperature or other unexpected damage, periodic calibration procedures can be carried out according to the actual situation and the system errors $\varphi_{\mathrm{SPE}}^{\mathrm{Monitor}}$ and $\varphi_{\mathrm{SPE}}^{\mathrm{Measure}}$ can be updated to improve the robustness of the system.

### 3.3. Accuracy Experiment

In the accuracy experiment, the SUT was a static surface with defocus and astigmatism generated by PDM. This static SUT was measured by DFMD according to the measurement strategy described in Section 2.1 and was measured by Zygo interferometer. The measurement results of DFMD and Zygo interferometer were compared to verify the measurement accuracy of DFMD.

**Figure 9:**
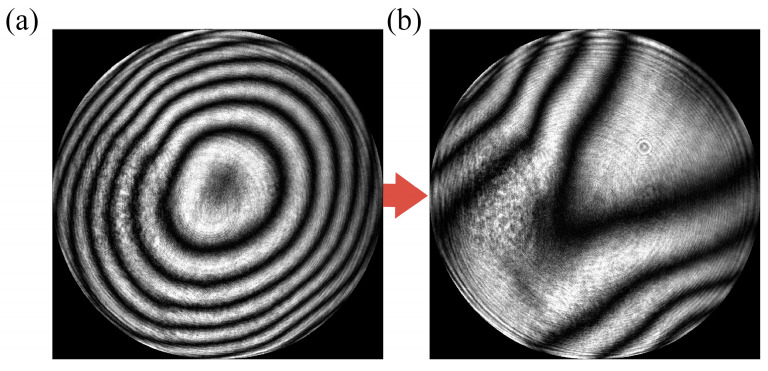
Interferogram obtained by PCMOS_1_ (**a**) before and (**b**) after phase modulation.

The phase modulation can be illustrated by changes in the interferogram of the measurement configuration (PCMOS_1_). Before the measurement started, the DM’s surface was set to the initial surface, which is close to a plane. Figure 9a,b show the interferogram obtained by PCMOS_1_ before and after modulation, respectively. Obviously, the interference fringes change significantly from dense to sparse. This phenomenon indicates that phase modulation is effective.

**Figure 10:**
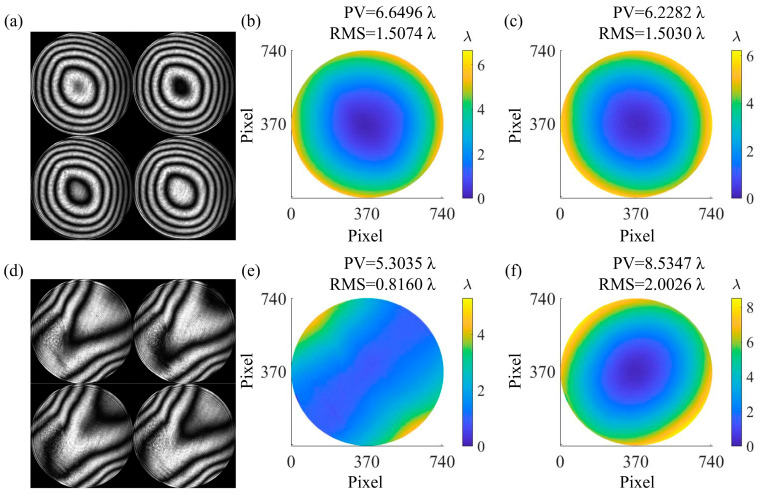
Calculation result of $\varphi_{\mathrm{SUT}}$. (**a**) Phase-shifting interferograms obtained by PCMOS_2_; (**b**) phase map of monitoring configuration $\varphi_{\mathrm{Monitor}}$; (**c**) phase map of $\varphi_{\mathrm{DM}}$ generated by the DM; (**d**) phase-shifting interferograms obtained by PCMOS_1_; (**e**) phase map of measurement configuration $\varphi_{\mathrm{Measure}}$; (**f**) phase map of $\varphi_{\mathrm{SUT}}$ generated by the static SUT.

$\varphi_{\mathrm{SUT}}$ was calculated from the interferogram obtained by PCMOS_1_ and PCMOS_2_ based on Equations (5) and (6). Figure 10a shows the interferogram of monitoring configuration obtained by PCMOS_2_, and Figure 10b shows the phase map of $\varphi_{\mathrm{Monitor}}$ with a PV of 6.6496 λ and an RMS of 1.5074 λ. The phase map of $\varphi_{\mathrm{DM}}$ is shown in Figure 10c, which has a PV value of 6.2282 λ and an RMS value of 1.5030 λ. Figure 10d shows the interferogram of measurement configuration obtained by PCMOS_1_, and Figure 10e shows the phase map of $\varphi_{\mathrm{Measure}}$ with a PV of 5.3035 λ and an RMS of 0.8160 λ. The phase map of $\varphi_{\mathrm{SUT}}$ is shown in Figure 10f, which has a PV value of 8.5347 λ and an RMS value of 2.0026 λ.

**Figure 11:**
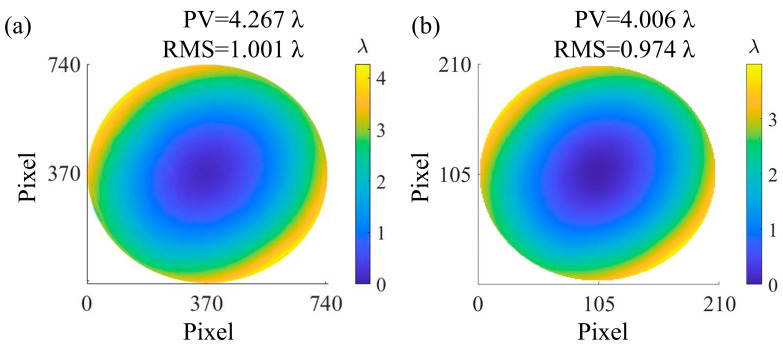
Surface figure error measurement results of (**a**) DFMD and (**b**) Zygo interferometer.

The surface figure error measurement results of DFMD and Zygo interferometer are shown in Figure 11a and Figure 11b, respectively. PV values are 4.267 λ and 4.006 λ, and RMS values are 1.001 λ and 0.974 λ, respectively. The deviations of PV and RMS are 0.261 λ and 0.027 λ, respectively, and the surface figure error map tends to be consistent. The comparison indicates that the PV accuracy and RMS accuracy of DFMD can reach 112 nm and 5 nm, respectively.

### 3.4. Dynamic Experiment

In the dynamic experiment, the dynamic SUT was a series of surfaces with continuously changed shapes generated by PDM, which is consistent with practical application. It included 10 surfaces with surface figure errors that first decreased and then increased. This dynamic SUT was measured according to the dynamic measurement strategy.

The phase modulation in the dynamic measurement can be illustrated by comparing the interferogram of the measurement configuration (PCMOS_1_) and the monitoring configuration (PCMOS_2_). The interferograms corresponding to the polarization direction 0° obtained by PCMOS_1_ and PCMOS_2_ are shown in Figure 11.

**Figure 12:**
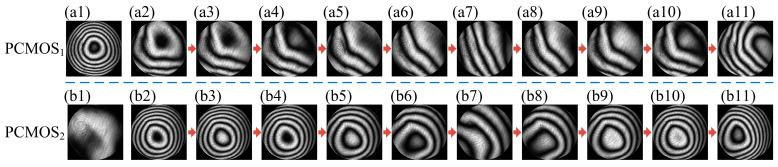
Interferogram corresponding to the polarization direction 0° obtained in the dynamic experiment. Interferogram obtained by PCMOS_1_ (**a1**) before and (**a2**–**a11**) during the dynamic measurement, interferogram obtained by PCMOS_2_ (**b1**) before and (**b2**–**b11**) during the dynamic measurement.

Figure 12(a1,b1) are the interferograms obtained by PCMOS_1_ and PCMOS_2_ before dynamic measurement, respectively. Phase modulation was not carried out at this stage. The interference fringes of the measurement configuration are dense, and the interference fringes of the monitoring configuration are relatively sparse. Figure 12(a2–a11) and Figure 12(b2–b11) were obtained by PCMOS_1_ and PCMOS_2_ during the dynamic measurement, respectively. Phase modulation was carried out at this stage. The interference fringes of the measurement configuration remain sparse. This indicates that the phase modulation target was realized. The interference fringes of the monitoring configuration change from dense to sparse, and then back to dense again. This phenomenon is consistent with the phase modulation law, that is, the density of interference fringes of the monitoring configuration is proportional to the phase provided by DM, and the phase provided by DM is proportional to the surface figure error of SUT, which first decreases and then increases. The analysis of Figure 12 indicates that the DM surface control neural network model realized phase modulation in the dynamic measurements.

**Figure 13:**
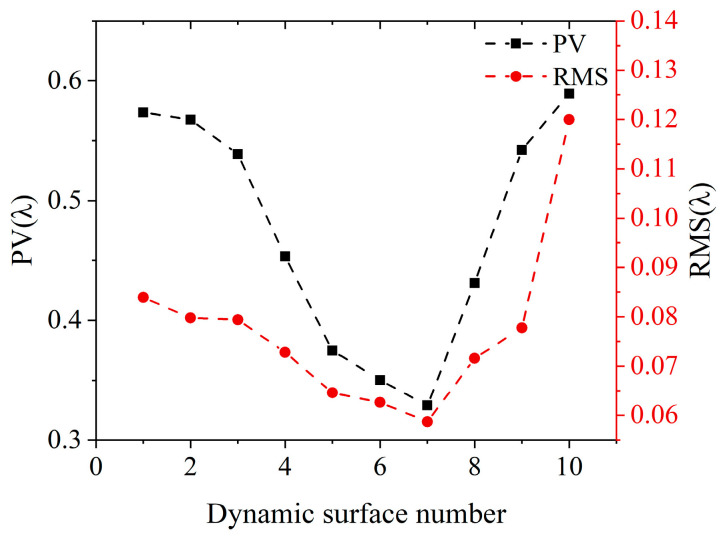
PV and RMS of the difference in sag between the DM target surface and the DM actual surface during dynamic measurement.

In the dynamic interferometric system, the beam is reflected once by the DM, so the phase modulation range is twice the deformation range of the DM. The effective aperture of DM in the experimental system was 20 mm, and the corresponding deformation was less than 8.8515 λ. Therefore, the phase modulation range was less than 17.7030 λ. The accuracy of phase modulation can be demonstrated by the PV and RMS of the difference in sag between the DM target surface and the DM actual surface during dynamic measurement, which is shown in Figure 13. The black dashed line is the PV curve, and the red dashed line is the RMS curve. The DM actual surface was generated based on the predicted control voltage vector output from the DM surface control neural network model. In Figure 13, the maximum values of PV and RMS appear in the measurement of the last surface, with PV and RMS of 0.5893 λ and 0.1210 λ, respectively. The minimum values appear in the measurement of the seventh surface, with PV and RMS of 0.3289 λ and 0.0587 λ, respectively. Therefore, the PV and RMS of the difference between the DM target surface and the DM actual surface can reach 208 nm and 37 nm, respectively.

**Figure 14:**
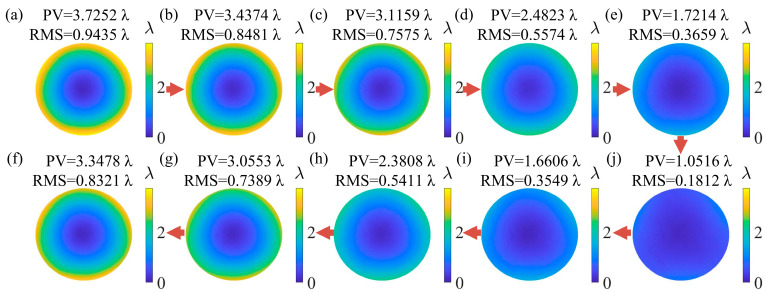
Figure error maps (**a**–**j**) corresponding to 10 surfaces.

**Figure 15:**
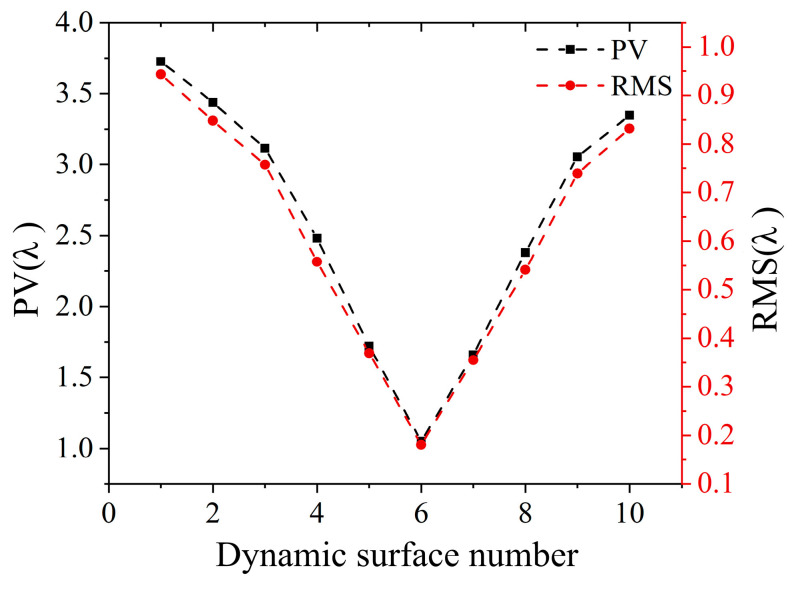
PV and RMS variation curves of surface figure errors for 10 surfaces.

The surface figure error measurement results were obtained. Figure 14 shows the surface figure error maps, and the corresponding variation curves of PV and RMS are shown in Figure 15. The black dashed line is the PV curve, and the red dashed line is the RMS curve. According to Figure 14 and Figure 15, the distribution of surface figure errors of the 10 surfaces is similar, and their PV and RMS both continuously decrease first and then increase. The measurement results are consistent with the SUT generated by PDM. Thus, DFMD realized dynamic SUT measurement.

### 3.5. Dynamic Response

The feasibility of accuracy and the dynamic of DFMD were verified by the experiments carried out in Section 3.3 and Section 3.4, respectively. In these experiments, the factors affecting the dynamic response include the following:

(1) Hardware response. The responding hardware in the experiment includes two PCMOSs and a DM. In this paper, the frame rate of PCMOS is 75 frames per second, and the response frequency of the DM is 70 Hz. In Section 3.3 and Section 3.4, the hardware response time reached milliseconds.

(2) Fitting speed of DM surface. Fitting is used to calculate the DM target surface vector for phase modulation. The sampling points of the DM target surface were fitted by the first 153 terms of the Zernike standard polynomial. The fitting involves large matrix calculation, which is the key factor affecting the fitting speed. The resolution of the experimental system is contradictory to the fitting speed, because the higher the resolution, the larger the amount of data in the DM surface fitting calculation, which leads to a decrease in the fitting speed. For the measurement requirements of large apertures, it is necessary to balance resolution and fitting speed according to the actual situation. In Section 3.3 and Section 3.4, the fitting time was limited by the computational performance of the personal computer adopted in the experiment. The fitting time can reach milliseconds by using a high-performance computer.

(3) Calculation speed of neural network model. The calculation speed is embodied by the time required for the neural network model to obtain the predicted control voltage vector according to the DM target surface vector. In Section 3.3 and Section 3.4, the calculation time reached milliseconds by using the CPU in the Python version 3.8 language environment. If the high-performance GPU is used in a C++ language environment by adopted Visual Studio 2024 developed by Microsoft, the calculation time can reach within milliseconds.

(4) Communication time. The hardware is controlled by software through communication. In Section 3.3 and Section 3.4, the communication between the software and PCMOS and DM was not integrated into the same language environment, which lengthens the communication time. By integrating into the same language environment, such as C++ provided by Visual Studio 2024, the communication time can reach milliseconds.

The analysis of factors affecting dynamic response indicates that the response time of DFMD can reach milliseconds to meet dynamic requirements when high-performance equipment is adopted, and the calculation and communication are integrated into the same language, such as C++.

## 4. Conclusions

We propose a dynamic interferometry for freeform surface based on machine learning-configured DM and named it DFMD. In DFMD, a DM is adopted to provide dynamic phases. To meet the requirements of dynamic and accuracy, a dynamic interferometric system with DM monitoring is designed and a transient phase modulation scheme based on machine learning-controlled DM is proposed, and measurements can be carried out based on the measurement strategy that combines the two. In this dynamic interferometric system, the coaxial structure and polarization phase shifting are used to simultaneously realize SUT transient measurement and DM transient monitoring. In this phase modulation scheme, the target phase is transiently obtained and converted into a DM target surface vector as the input of the DM surface control neural network model. The voltage vector output by the neural network model is used to control the DM to generate the corresponding surface to realize the target phase transiently. DFMD is verified by practical experiments. The DM surface control neural network model is constructed and tested, and its control accuracy can reach nanometers in both PV and RMS. The interferometric experimental system is built and the system errors are calibrated. The measurement results of static SUT and dynamic SUT verify the feasibility of accuracy and dynamic, respectively. The factors affecting the dynamic response are analyzed.

In the future, we plan to further improve DFMD and apply it to the manufacture and assembly of freeform surfaces. For example, optimizing the optical structure to expand the phase modulation range of DM to provide possibilities for complex freeform surface measurements; optimizing the neural network model by enriching the diversity of DM surface samples, which can be realized by loading randomly generated PZT control voltage. In the experiment of this paper, the SUTs have a satisfactory reflectivity. In the future, when it is applied to in-line measurement, if the SUT reflectance undergoes a reduction due to manufacturing materials or other reasons, an attenuator can be added to the system to obtain acceptable interference fringe contrast.

## Data Availability

Data are contained within the article.
